# Society of Physicians and Surgeons

**Published:** 1878-04

**Authors:** Junius M. Hall


### Society Reports.

### SOCIETY OF PHYSICIANS AND SURGEONS.

REPORTED BY JUNIUS M. HALL, M. D.

Regular meeting at Grand Pacific Hotel, March 11th, 1878, President Wm. H. Byford, M. D. in the chair.

The minutes of the last meeting were read and accepted by the Society. Dr. Henry W. Boyd was elected a member of the Society.

The following paper on EXFOLIATION OF THE COCHLEA was then read by Dr. S. O. RICHEY.

This process is a comparatively rare one, and is one of the results of chronic suppurative inflammation, which in its course involves the labyrinth.

The membrana tympani has generally been destroyed, and the ossicles of the middle ear have previously been lost. Sometimes it has been complicated with polypi and mastoid abscess. In many of the cases observed, the cochlea alone of the labyrinth has been thrown off, while in other cases the cochlea, vestibule, semi-circular canals, etc., have been exfoliated.

It has been claimed that some power of audition has remained after the cochlea alone has been removed, but that when the whole labyrinth has been destroyed no hearing power has remained, while there has usually been facial paralysis.

It is difficult to decide, without an autopsy, that no part of the labyrinth except the cochlea has been lost, unless the surgeon is present, each time the ear is syringed, so that he may know what is cast off when he sees it. It is well known that the ossicles are often washed out by the patient or his friends without being seen by them. May not a sequestrum from the labyrinth as easily escape notice?

Facial paralysis accompanying chronic suppurative inflammation of the tympanum, is not a reliable symptom of the destruction of the entire labyrinth, though it be associated with complete deafness, as it may arise from other causes.

I have known one case in which partial paralysis of the right portio dura seemed to be due to the pressure of accumulated pus in suppurative inflammation of the right middle ear. There was no reason to suppose the labyrinth was at all affected, as the hearing was improved by removing the pus from the middle ear, and the paralysis was relieved when the discharge ceased. It has been my good fortune to have under observation at the Illinois Charitable Eye and Ear Infirmary, two cases in which the cochlea became necrosed, and was extracted.

The first case is that of Daniel Gubbins, aged 8 years, who came under care as a dispensary patient, May 30th, 1876. The statement of the mother of the boy is, that more than 8 months prior to this time, the boy had been sick with measles, and during his sickness, " he had a bad ear ache " in the right ear, which " got very red and swelled up."

A day or two afterwards " he had a gathering " in that ear. She claimed to have washed several " gravels " from the right ear, which she thought might have been the cause of the inflammation. These were in all probability the ossicles of the middle ear.

At the time of his application for relief, the tympanum contained fetid pus, which escaped from the external meatus, and through two other openings, one above, and the other behind the auricle. The pus escaping from the fistula above the auricle had burrowed under the retrahens muscle, and had formed a sinus at least an inch in length. Necrosis of the squamous portion of the temporal bone had occurred at the upper opening, about which, in consequence, there was unhealthy and excessive granulation.

Several spiculae of dead bone were taken from this region, and the granulations were reduced by the solid nitrate of silver. The membrana tympani was entirely destroyed, and the external meatus and middle ear contained numerous polypi, which were extracted, and their bases cauterized. Medicated solutions could then be forced into the meatus and out through the artificial openings, or into these openings and out through the meatus.

The whole right side of the head seemed enlarged, and the child staggered if he attempted to walk alone; the mother steadied him by keeping her hand on his shoulder.

Even after he had improved greatly, if he endeavored to cross the room alone to a chair on the opposite side, he would go to a point somewhat to the left of the chair, until he came near it, when he would stop, change his deviation and reach the chair.

Instead of carrying his head erect, he leaned it very much towards the left shoulder.

If he were asked a question, he would after some hesitation, answer in a quick jerking manner. He was pallid, slept badly, and had a poor appetite, but he did not complain of any pain at this time.

Hearing distance, right ear$=\frac{0}{48}$.

Profuse granulation took place in the sinus behind the auricle, and continued, regardless of the efforts made to control it, until it became necessary to open up the sinus.

In doing this the retrahens muscle had to be cut across, and of course the auricle fell forwards and below the plane of the other auricle, giving to the patient a somewhat peculiar appearance. The cicatrix, by contraction, has restored the pinna almost to its former position. The improvement has been gradual, and although the suppuration has nearly ceased many times, it has been again brought on by inattention at home and by exposure. The treatment has been cleanliness, and the application of carbolized and astringent solutions, the astringent being changed from time to time.

A strong solution of the nitrate of silver has been applied once a week.

Sept. 15th, 1877.— The necrosed cochlea was removed from the middle ear in which it could be seen. It is nearly perfect in form. At no time has there been any evidence of facial paralysis, so far as has been observed, nor does there seem to be any power of audition on the right side.

Every sound he recognizes he attributes to the left ear, even though the meatus of that side is closed.

The *second case* is that of Mr. Bernhard Sturm, aged 40 years, a resident of Jefferson county, Ill., who became an inmate of the Illinois Charitable Eye and Ear Infirmary, May 19th, 1877. His history, as given by himself, is as follows:

"He does not know that he had any trouble with his left ear prior to his tenth year. At that time, while standing on ice, he was thrown down and his head struck the ice with so much force that he was insensible for a day and a night afterward, and he does not think he has heard anything with his left ear since." In the cranium at the junction of the sagittal with the lambdoidal suture, there is a depression so deep as to receive a finger, its length being in the direction of the lambdoidal suture. This he claims to be a result of the fall. Subsequent to the fall, he felt no inconvenience except as to the loss of hearing in the left ear, until eight years ago, when it began to have a disagreeable odor, and continued to do so, although no discharge was noticed until the middle of March, 1877.

During the two winters preceding that of 1876-7, he had constant and severe pain in the left ear, which caused him much loss of sleep. This pain left him each spring when the weather became warm, until 1877. The left ear was unusually painful immediately before it began to discharge, March 1877. From the time suppuration began until he came to the infirmary, he could obtain only one or two hours' sleep in the twenty-four. He claims to have been several times "out of his head" with pain. For many weeks before he came into the infirmary, he could not walk alone because of dizziness, and if he attempted to turn around quickly, he would fall. Dr. J. H. Newton, his family physician, writes in regard to his case: "About March 12th, 1877, after a few days' exposure to a cold rain, he was taken with intense pain in his head and ear, accompanied with rapid pulse, vomiting and delirium. Upon the subsidence of the first fever, diplopia showed itself, and remained with him while he staid here, together with unsteady gait and confusion of ideas. Somewhere about May 1st, a small piece of bone, very much corroded, came out of his ear. I thought it a portion of the malleus."

The patient also mentioned this piece of bone, and compared it to a grain of wheat in size and shape.

When he was admitted into the infirmary, he had severe pain all through his head, referable particularly to the left temporal region. His appetite was poor, and he complained of constant nausea. His countenance was pallid, his gait unsteady, and he weighed 125 pounds. There was diplopia, due to the divergence of the left eye, and the vision of that eye was somewhat confused. Nothing was done for this directly, but the difficulty disappeared in about one month, under the treatment to which the ear was subjected. In the external meatus, near the membrane of the drum, growing from the anterior and upper portion of the canal and nearly filling it, was a polypus.

No portion of the membrane of the drum remained, the ossicles had been lost, and the middle ear contained several polypi, bathed in pus. These polypi were removed with forceps, and their bases destroyed with chlor-acetic acid. Hearing distance, left ear=$\frac{0}{48}$.

Two days later the meatus was so swollen that it would admit only a small probe with a little cotton twisted tightly about it. By this means nitrate of silver was applied to the external meatus and the middle ear, and the swelling very gradually reduced. The pain in the head continued, except when relieved by blisters over the mastoid process, by bromide of potassium, or by quinine, until the first sequestrum, a portion of the cochlea, was removed, Oct. 19th, '77.

Suppuration also continued freely during this period, after which it immediately diminished, until it was almost imperceptible, and the patient was free from pain.

The external meatus slowly resumed its natural size, the patient slept comfortably, his appetite improved, and he gained in weight until Nov. 20th, when he weighed 153 pounds, three pounds more than his usual weight.

Nov. 20th. A second sequestrum was taken from the middle ear. I had touched it and turned it over two days before with a probe, and it seemed then to be buried in the soft tissue, in the posterior portion of the tympanum, near the mastoid cells. The day it was removed, it could be seen, beyond the middle ear, and was withdrawn by a small hook. Its shape and structure indicate that it was thrown off from the mastoid cells. Since that time no suppuration has occurred in this ear, nor has there been any pain in it.

Dec. 29th.—A new membrane of the drum was recognized by Dr. S. J. Jones, occupying the usual position of the membrana tympani, but lacking of course the support of the ossicles. It was less concave externally than is common.

When the middle ear was inflated the membrane could be seen to move outward. It would resume its original shape and position when the air was withdrawn from the middle ear.

Bromide of potassium diminished the pain in the head for a short time, early in the treatment of the case, and afterwards seemed to be useless. Sulphate of quinine was substituted for it, and relieved the prominent symptom. The patient had lived in a miasmatic district, and the presence of a malarial element is probable. The watch cannot be heard when pressed upon the left auricle. If one tip of the diagnostic tube be placed in the left external meatus, and a vibrating tuning fork be placed in contact with the other end of the tube, he does not hear it. If a vibrating tuning fork be placed on any portion of the cranium, he always attributes the sound to the right ear. The voice is heard with much more difficulty, if both meati are occluded, than if the right meatus alone be closed. Thus the evidence of power of audition in the left ear is entirely negative in its character.

That there is hearing power in this ear may be possible, but if so, it is too small to be of any practical value.

Each of these cases presents some points of special interest. They are similar in the unsteadiness of gait, having a tendency in direction toward the healthy side.

The first sequestrum, in the case of the man, is smaller, more imperfect, and was more difficult of removal than that of the boy, whose cochlea is nearly normal in shape. The boy had mastoid abscess, with free communication between the mastoid abscess, the external meatus, and the pharynx. Parts of the temporal bone were lost by necrosis, and he leans his head toward the shoulder of the healthy side.

In the case of the man, pain in the head existed for a period of several months, and there was diplopia, due probably to some interference with the healthy action of the third nerve, which supplies motion to the internal rectus, or of the sixth nerve, which performs the same office for the external rectus.

The treatment has been much the same in each case, nitrate of silver being the chief application locally.

Dr. Richey then exhibited the specimens of necrosed bone removed from the two patients. At his request both of the patients were present, and an opportunity was given each member to examine them per speculum.

Considerable discussion followed.

Dr. Montgomery had listened with great interest to the report of the two cases by Dr. Richey. He had never met with a case nor had he seen mention of a case where the membrana tympani had been restored after exfoliation of the cochlea. It was certainly a very rare result.

In acute suppuration with a tendency to mastoid complication, he had had very good results from the use of the tincture of iodine as a counter irritant, applied all around the auricle. Internally the parts should be kept thoroughly cleansed, and astringents and stimulants occasionally applied. Nitrate of silver is his favorite application.

Dr. Bevan related one or two cases to show the great mental depression and lack of self confidence in those who had suffered for any length of time from serious ear trouble.

Dr. S. J. Jones reiterated the sentiments of Dr. Bevan. He considered it very unfortunate for the patient, that he should allow his disease to continue for so long a time, before applying to the surgeon for relief. If application were only made when the disease first showed itself, the hearing would undoubtedly be greatly benefited in the majority of cases.

Dr. S. J. Jones presented three additional cases in which there had been closure of artificial openings in the membrana tympani, the result of spontaneous perforation, occuring as the result of suppurative inflammation of the middle ear. They presented different aspects of the drum membrane after such closure, in all of which the new membrane formed, seemed to be thinner than the normal membrane of the drum. They showed how incorrect is the general impression, that any opening in the drum membrane is permanent and that it is fatal to hearing. Allusion was also made to another fallacious impression that it is dangerous to check a suppurative process of long standing in the ear, lest metastasis occur. This discharge being a consequence of an inflammatory process, must find an exit, and it usually does so though the drum membrane and occasionally, also, though the mastoid process. There can be no danger in controlling the inflammation which is producing this discharge and destruction or alteration of the tissues locally, and which often impairs the general health.

The course of treatment usually pursued in these cases is thorough cleansing of the parts as of primary importance, and following this with stimulants and astringents. Strong solutions of nitrate of silver, and also tincture of myrrh are serviceable.

A case of double congenital coloboma of the iris (in a child two years old) was also presented at the meeting. In the right eye the coloboma is upwards and outwards, whilst in the left eye it is downwards and outwards. There is present alternating convergent strabismus, but the left eye is more relied upon by the child for vision than is the right one. There is no evidence of a similar affection in any other member of the parents' families.

The history of three similar cases of coloboma in one family which had recently come under Dr. Jones' notice was also given. Three out of eight children were thus affected. The opening being not only through the iris but through the choroid as well, so that the sclerotica could be seen with the ophthalmoscope, almost to the entrance of the optic nerve. Their ages are respectively ten, five and two years. Children born between these had no such defect, nor could any information be obtained of any similar cases in either of the parents' families, nor of any hare-lip or of cleft palate.

The meeting then adjourned.

